# The rs2682826 Polymorphism of the *NOS1* Gene Is Associated with the Degree of Disability of Erectile Dysfunction

**DOI:** 10.3390/life13051082

**Published:** 2023-04-25

**Authors:** Leticia Perticarrara Ferezin, Cezar Kayzuka, Vitória Carolina Rondon Pereira, Murilo Ferreira de Andrade, Carlos Augusto Fernandes Molina, Silvio Tucci, Jose Eduardo Tanus-Santos, Riccardo Lacchini

**Affiliations:** 1 Department of Psychiatric Nursing and Human Sciences, Ribeirão Preto College of Nursing, University of Sao Paulo, Ribeirao Preto 14040-902, Brazil; 2 Department of Pharmacology, Faculty of Medicine of Ribeirao Preto, University of Sao Paulo, Ribeirao Preto 14049-902, Brazil; 3 Department of Surgery and Anatomy, Ribeirao Preto Medical School, University of Sao Paulo, Sao Paulo 05508-090, Brazil

**Keywords:** erectile dysfunction, polymorphism, nitric oxide, neuronal nitric oxide synthase, rs41279104, rs2682826, rs2389866, rs3733526, rs13124532

## Abstract

**Simple Summary:**

As age advances, the risk for developing erectile dysfunction (ED) in men increases. ED can be a mark for cardiovascular diseases, as well as be related to mental disorders such as depression. There are some inherited features that can predict such complications. Therefore, in this study, we investigated whether the variation in the gene that produces the enzyme called nNOS in the body, which is important for maintaining cardiovascular balance, may be related to the risk for the development of ED or the severity of ED. We found that a variant of the *nNOS* gene may be related to a worse degree of ED. This means that those who have a variation in this region of gene may have a worse degree of ED. Despite the need for confirmation of these data, perhaps these genes can contribute to predicting the risk of this population in developing severe degrees of ED.

**Abstract:**

Erectile dysfunction (ED) is a common male disorder, often associated with cardiovascular disease and ageing. The Sildenafil, a PDE5 inhibitor, can improve the erectile function by prolonging the nitric oxide (NO) downstream effect. NO is a molecule of pivotal importance in erection physiology and is mainly produced by neuronal nitric oxide synthase (nNOS) and endothelial NO synthase (eNOS). While it has been shown that *eNOS* and *nNOS* genetic polymorphisms could be associated with Sildenafil responsiveness in ED, no study so far has assessed whether nNOS polymorphisms and *PDE5A* polymorphism could be associated with increased risk to ED or with intensity of symptoms. A total of 119 ED patients and 114 controls were studied, with evaluation of the clinical disability by the International Index for Erectile Function instrument, plasma assessment of nitrite levels and genomic DNA analysis regarding the rs41279104 and rs2682826 polymorphisms of the *NOS1* gene and the rs2389866, rs3733526 and rs13124532 polymorphisms of the *PDE5A* gene. We have found a significant association of the rs2682826 with lower IIEF scores in the clinical ED group. While this result should be confirmed in other populations, it may be helpful in establishing a genetic panel to better assess disease risk and prognosis on ED therapy.

## 1. Introduction

Erectile dysfunction (ED) is a multifactorial condition, characterized by the inability to obtain or maintain an erection sufficient for satisfactory sexual intercourse [1,2,3], and its main cause is vascular, although other causes such as endocrine and physical psychological are also important [1,2]. In general, the tests commonly used to assess the diagnosis of ED include cholesterol and blood glucose tests, which evaluate risk factors for the disease, and imaging tests—especially the penile doppler ultrasound, which provides important information about the condition of the penile blood vessels [2,3]. Another way to assess the presence and degree of erectile dysfunction is applying the IIEF (International Index of Erectile Function), a questionnaire with fifteen items and five domains of sexual function (erectile function, orgasmic function, sexual desire, sexual satisfaction and sexual satisfaction) [4,5]. Item scores range from zero or one to five [4,5]. The lower the score on each item, the worse the function studied, meaning that patients with more serious degrees of ED have lower scores [4,5].

There is a strong relationship between ED and cardiovascular diseases (CVDs), which involves different risk factors and common pathophysiology, such as endothelial dysfunction, inflammation and low plasma testosterone levels [1,6]. Hypertensive individuals or people with other CVDs have a higher risk of developing ED compared to healthy individuals [6], and ED is considered a risk marker for future CVDs [6,7,8,9].

Although this is a very common disease in older men, there are individuals who are affected earlier than others or whose symptoms are more intense than other individuals of the same age group [10,11]. This could be possibly explained by an interaction between environmental factors such as smoking and alcoholism, preexisting conditions such as endocrine and mental illness, and genetic polymorphisms, especially Single Nucleotide Polymorphisms (SNPs), which may predispose a subject to develop such a condition [12].

Under these conditions, a very important route that is affected by genetic polymorphisms is the nitric oxide pathway [13]. Its main source is enzymatic, with neuronal nitric oxide synthase (nNOS, or NOS1) being expressed in non-adrenergic non-cholinergic nerve fibers (NANC) [14], while endothelial NO synthase (eNOS, or NOS3) is expressed in endothelial cells [15]. Both enzymes play a key role in the onset and maintenance of the penile erection [16,17]. After sexual stimuli, the NANC fibers release the first production of NO, which is later complemented by other sources, namely the enzyme endothelial nitric oxide synthase (eNOS) in the corpora cavernosa [15].

The main mediator involved in erection is nitric oxide, released by the endothelium and parasympathetic nerve endings [1,6,7]. Briefly, the relaxation of the cavernous smooth muscle leads to engorgement of the sinusoids (what means that these structures are filled with blood) and causes compression of the subtunic veins between the trabeculae and the tunica albuginea, causing occlusion of the venous return, which culminates in the erection [1,2,3,6].

The nitric oxide produced by the NO synthases rapidly diffuses into the vascular lumen and into the smooth muscle cell, where it interacts with the iron in the heme group of the enzyme soluble guanylate cyclase (GCs), activating it [18]. Active soluble guanylate cyclase catalyzes the release of two phosphate groups from the guanosine triphosphate (GTP) molecule, culminating in the synthesis of cyclic guanosine monophosphate (cGMP) [18]. Increased concentration of cGMP in the smooth muscle cell causes relaxation, which basically involves decreasing Ca^2+^ entry into the cell, inhibiting Ca^2+^ release from the sarcoplasmic reticulum and increasing Ca^2+^ sequestration to the sarcoplasmic reticulum [19].

The end of the NO signaling occurs through the degradation of cGMP by enzymes called phosphodiesterases (PDEs) [20,21]. There are different subtypes of PDEs, and each one is selective for cAMP (cyclic 3′,5′-adenosine monophosphate, a substance produced in a reaction catalyzed by the enzyme adenylate cyclase), cGMP, or both [20,21]. PDEs, which act as regulators of cGMP levels, have so far been identified in eleven isoforms [20,21]. In terms of function, the most relevant PDE isoform in human penile tissue is PDE5, specific for cGMP [20,21,22]. Based on the advantages of its use form (oral) and clinical efficacy, the most prescribed treatment (and the first line pharmacological attempt) for erectile dysfunction is the oral PDE5 inhibitors, such as sildenafil, tadalafil, vardenafil, and avanafil [2,3].

Some research found out that variations on the *PDE5A* may be clinically relevant. A genome-wide association, for example, found that a single-nucleotide polymorphism (SNP) in the *PDE5A* gene (rs2389866, a C/T single-nucleotide variation) is one of the top five SNPs associated with brachial artery endothelial function traits [23]. Other research found that rs13124532 (a C/T single-nucleotide variation) was associated with all of the following: the susceptibility to Immunoglobulin A nephropathy (IgAN, the most common form of chronic glomerulonephritis GN in pediatric patients), and the development of proteinuria, podocyte foot process effacement and pathological progression [24].

The nNOS, the important enzyme responsible for the onset of erection, is encoded by the *NOS1* gene [25], which has functional polymorphisms, including rs41279104 [13,26,27] and rs2682826 [28,29]. Polymorphisms of this enzyme have also been associated with an altered response to sildenafil in ED [13] and the increased risk of psychiatric illnesses such as schizophrenia [30,31], depression [32] and suicidal behavior [29]. Furthermore, other studies on genes that directly impact the nitric oxide pathway have shown associations of SNPs with an increased risk of erectile dysfunction and poor response to Sildenafil [12,33,34,35].

Thus, it is possible that genetic markers on *NOS1* and *PDE5A* may help to explain the natural vulnerability to the onset of NO pathway imbalances that culminate in a greater risk of developing erectile dysfunction and having more severe degrees of this disease.

The aim of this study was to assess whether two *NOS1* genetic polymorphisms (rs41279104 and rs2682826) and three *PDE5A* genetic polymorphisms (rs2389866, rs3733526 and rs13124532) are associated with the risk of ED and the degree of dysfunction.

## 2. Materials and Methods

This is a case-control, cross-sectional, strictly observational and prospective study. It was performed according to the Declaration of Helsinki, submitted to our institutional Ethics Committee, and approved under the number CAAE (51408515.9.0000.5393). All included participants signed the informed consent form. 

**Figure 1:**
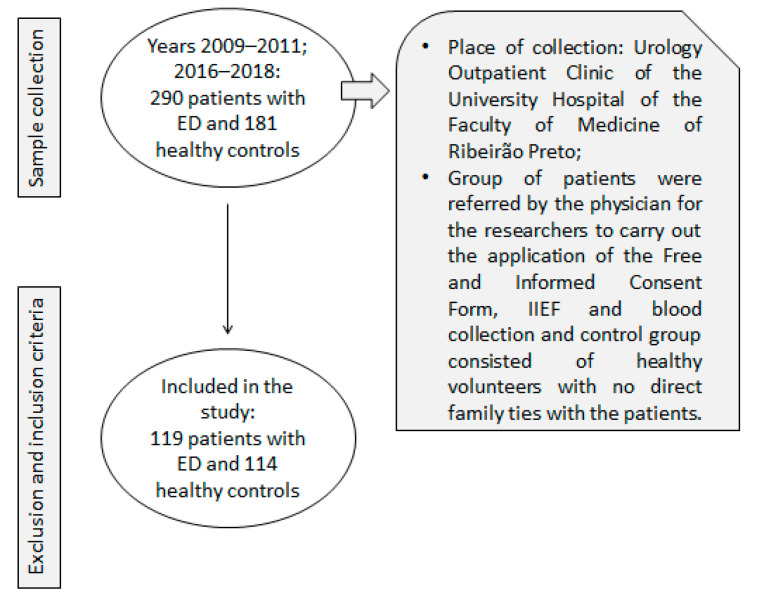
Flowchart showing of patients and controls selection.

The participants included (Figure 1) were patients with ED (n = 119) from the Urology Outpatient Clinic at the University Hospital of Ribeirao Preto Medical School and healthy controls (n = 114), who were passers-by individuals in the university campus community. The inclusion criteria for both groups were to be aged between 18 and 80 years. Specifically for the Patient Group, complaints of sexual activity and medical diagnosis of erectile dysfunction were also considered. Exclusion criteria for both groups were: psychiatric, neurological, endocrine, cardiovascular, anatomic disorders or disabilities (for example cancer, history of cardiac arrest, hypogonadism, endocrine tumors, spinal cord injury, Peyronie’s disease, prostatectomy, schizophrenia, major depressive disorder, suicide attempts, etc.). Clinical phenotyping of ED included the application of the International Index of Erectile Function (IIEF), a questionnaire with fifteen items and five domains of sexual function (erectile function, orgasmic function, sexual desire, sexual satisfaction and general satisfaction). Item scores ranged from zero or one to five [4,5] to quantify the volunteers’ degree of disability. The lower the score on each item, the worse the function studied, in other words, patients with more serious degrees of ED have lower scores [4,5]. Participants of the control group with an IIEF score less than 22 were excluded, and participants of the ED group with an IIEF score equal to or greater than 22 were excluded. 

Genotypes for all polymorphisms of the *NOS1* and *PDE5A* genes were determined as previously described [13]. At the time of blood collection, part of the samples were treated with sodium nitroprisside and centrifuged within 5 min after puncture of the forearm to separate the red blood cells from the plasma in order to perform nitrite measurements. In another part of the samples, DNA was extracted from the whole blood by salting, followed by purification with phenol–chloroform. DNA samples were diluted to 5 ng/µL and used in Taqman Genotyping assays. All assays were pre-designed by Life Technologies (Foster City, CA, USA; assay IDs: C_86363451_10 and C_15907244_10 for *NOS1* and C__15784934_10, C___239251_1_ and C__9553160_10 for *PDE5A*) and reactions used 5 ng input DNA, 1× GoTaq Probe qPCR master mix (Promega, Madison, WI, USA) and 1× Taqman assay. Thermal cycling and fluorescence acquisition were performed on a StepOne Plus qPCR machine (Life Technologies, Foster City, CA, USA).

Numerical variables were tested for normality and the parametric or non-parametric statistics was applied accordingly. Numerical variables were compared between case and control groups using Student’s t test or the Mann Whitney test. Numerical variables were compared between groups of haplotypes by ANOVA followed by Tukey’s post-hoc test or by Kruskal–Wallis followed by Dunn’s post-hoc test. Categorical variables or genotypes frequencies were compared between groups by chi-square tests. In addition, we performed a multiple linear regression model to assess the association between genotypes and the IIEF score correcting for confounding factors. This model was built by sequential analysis to identify factors that were associated in univariate and bivariate analyses. Final models included age, diabetes, smoking and ethanol consumption. All analyses considered *p* < 0.05 to be statistically significant.

In this study, we used two different software types to estimate our statistical tests power. To assess the case-control analysis power, we used PGA v.2.0 software [36,37]. To analyze the association of genetic markers with erectile function, we used the dominant inheritance model, the frequency of the disease estimated at 17% in the United States of America [7], the frequency of the smaller rs11119328 allele in our sample (11%), the total of 119 cases, the control-to-case ratio 0.96 (114 controls/119 patients), effective degrees of freedom 2.00 (calculated with our raw genetic data with EDF tool in the PGA package), power of 0.8 and alpha 0.05. We were able to detect associations with an odds ratio (OR) greater than 2.14 with a power of at least 80. We may have been underpowered to an OR below those mentioned above.

To calculate the power of multivariate linear regression analyses, we used G * Power v. 3.1.9.2 [38,39]. We had models with 12 predictors (independent variables), n = 142, r^2^ = 0.22 and alpha = 0.05. In this situation, power was calculated as 0.99.

## 3. Results

Table 1 presents clinical characteristics of the subjects included in this study (control group and clinical erectile dysfunction group). The groups were matched for ethnicity and body mass index. However, we were unable to control both groups by age, smoking, blood pressure, ethanol consumption, and other variables. The variables considered most important that were not paired between the two groups were included in the multivariate analyses, in order to increase reliability of the results. 

Supplementary Table S1 shows the genotypes and allele frequencies of rs41279104 and rs2682826 (*NOS1* polymorphism). We found no association of *NOS1* genotypes with ED, which was confirmed by multivariate logistic regression analysis (not shown; adjusted *p* values were 0.551 and 0.640 for rs41279104 and rs2682826, respectively).

**Figure 2:**
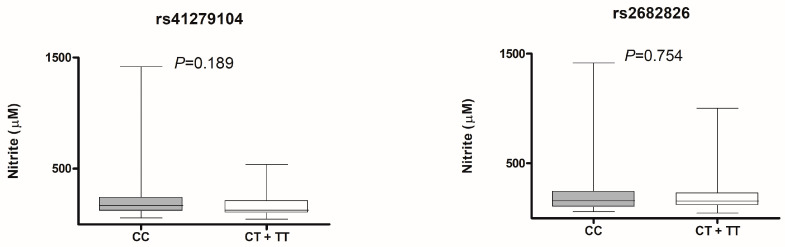
Nitrite plasma concentration versus genotypes of *NOS1* polymorphisms—Clinical ED group. Values of the nitrite concentration expressed as median ± interquartile ranges. Mann Whitney test.

Supplementary Figure S2 shows the IIEF scores of subjects with ED according to the *NOS1* polymorphisms. In the uncorrected analysis it was not possible to observe a significance (*p* = 0.109) between the rs2682826 and the erectile function evaluated by the IIEF in the clinical ED group. However, in the multivariate linear regression analysis (Table 2), we observed that the variant genotypes (CT + TT) were negatively associated with the IIEF score (*p* = 0.033, B = −0.07), indicating that a subject with clinical ED carrying CT and TT genotype of rs2682826 may present worse erectile function when compared to a CC genotype subject with clinical ED. Figure 2 shows no association between *NOS1* polymorphisms and nitrite levels in plasma, which was confirmed by multivariate linear regression (Supplementary Table S2). 

Supplementary Table S3 presents the haplotype frequencies of *NOS1* in the studied groups. We found no association between haplotypes and ED. Besides, there was no association between IIEF score and *NOS1* polymorphisms in the uncorrected analysis (Supplementary Figure S2), nor on multivariate analysis (Supplementary Table S4). Curiously, the TT haplotype was just observed in the clinical ED group.

The haplotypes of *NOS1* polymorphisms were not associated with plasma nitrite levels of the ED group (Supplementary Figure S3 and Supplementary Table S5).

Regarding the polymorphisms of the *PDE5A*, Supplementary Table S6 shows the genotypes and allele frequencies of *PDE5A* polymorphisms (rs2389866, rs3733526 and rs13124532) of the studied groups. We did not find an association of *PDE5A* genotypes with ED, which was confirmed by multivariate logistic regression analysis (not shown; adjusted *p* values were 0.510 and 0.454 and 0.909 for rs2389866, rs3733526 and rs13124532, respectively).

Supplementary Figure S4 shows the IIEF scores of subjects with ED according to the genotype of *PDE5A* polymorphisms. In the uncorrected analysis, it was not possible to observe a significance in the three polymorphisms of *PDE5A*. This observation was confirmed in the multivariate linear regression analysis (Table 3).

**Figure 3:**
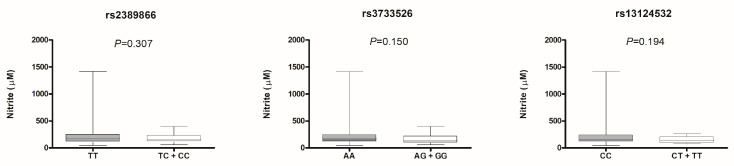
Nitrite plasma concentration versus genotypes of *PDE5* polymorphisms—Clinical ED group. Values of the nitrite concentration expressed as median ± interquartile ranges. Mann Whitney test.

Then we evaluated the influence of *PDE5A* polymorphisms on plasma nitrite levels, and we observed no associations in uncorrected analysis (Figure 3) nor in multivariate models (Supplementary Table S7).

Regarding *PDE5A* haplotypes, we found no association with ED (Supplementary Table S8 and multivariate logistic analysis (not shown; *p* = 0.989)). We also show that these haplotypes have no association with IIEF values (Supplementary Figure S5 and Supplementary Table S9). Besides, *PDE5A* haplotypes also did not associate with plasma nitrite levels (Supplementary Figure S6 and Supplementary Table S10).

## 4. Discussion

This is the first study showing an association between the rs2682826 polymorphism of the *NOS1* gene and the IIEF erectile function score (disease severity) in clinical ED patients. We found that for the rs2682826 polymorphism of the *NOS1* gene, the CC genotype in clinical ED patients, had a positive influence on the values IIEF score for erectile function, meaning that carriers of this genotype have better erectile function, while carriers of the CT + TT genotype demonstrated a negative association with this IIEF score, implying worse degree of erectile function (Table 2). However, with the rs41279104 polymorphism, we did not find any association with the risk of ED or the degree of severity. We found no associations of *PDE5A* genotypes and haplotypes with ED risk, IIEF scores or nitrite levels. 

NO has multiple functions throughout the human body, including a neurotransmitter-like function in the brain and peripheral nervous system, in addition to blood pressure regulation through endothelium-derived relaxing factor activity [1,17]. NO is synthesized by specific enzymes, the NO synthases (NOS), a family that includes three distinct isoforms: neuronal NOS (nNOS; encoded by the *NOS1* gene), inducible NOS (iNOS; NOS2 gene) and endothelial NOS (eNOS; NOS3 gene) [40,41]. Regarding erectile dysfunction in animal models, the importance of NO production via nNOS for maintaining penile erection has already been demonstrated [1,42]. 

The *NOS1* gene is located on chromosome 12q24.2 and is composed of 29 exons and 28 introns, comprising more than 160 kb of genomic DNA [12,41]. The rs2682826 polymorphism, where is observed an exchange of cytosine (C) for thymine (T), is located in the 3′UTR region of *NOS1*, in exon 29, which is considered critical regarding the conditions of expression of the *NOS1* gene and can produce differential transcripts through several different polyadenylation sites [25,43]. Furthermore, the 3′UTR region has a known role in the degenerative stability and translational efficiency of the mRNA, being considered functionally important [44].

Although there is only one paper in the literature showing the association of the rs2682826 polymorphism with erectile dysfunction in response to Sildenafil [13], in our work we found an association of this SNP (rs2682826) with a more severe ED degree. This means a lower function of the gene in individuals carrying the variant genotypes.

Corroborating the results found in our work, a study [28] that evaluated the association of the rs2682826 polymorphism with Achalasia (a disorder that is characterized by esophageal stiffening) showed that the variant genotype (TT) was more common in those affected by the disease than in healthy controls. NO is the main inhibitor of the esophageal myenteric plexus, meaning that it progressively delays muscle contraction in the esophagus, and in this context, carriers of the variant genotype of this polymorphism may have a lower function of the gene. In addition, other studies contribute to this argument, in which the variant allele of rs2682826 is associated with a lower function of the gene [29,45,46,47].

The rs41279104 (C>T) polymorphism is located at the promoter region of *NOS1* gene and proved to be functional, as a genetic alteration in the regulatory region 1c of the *NOS1* exon can influence gene expression. In this polymorphism, the variant T allele decreased nNOS expression by 30% [48]. Some studies show that the variant T allele was associated with several loss-of-function phenotypes reducing nNOS activity, namely infantile hypertrophic pyloric stenosis [48], cerebral malaria [49], cutaneous melanoma [50], reduced markers of central serotonergic activity [51] and schizophrenia [30,52,53]. 

People with schizophrenia who carry the T allele may also show a marked decrease in nNOS expression in the prefrontal cortex [54]. Therefore, this information already present in the literature suggests that the polymorphisms of our study may affect the levels of the nNOS transcripts in the studied participants. However, in the present work, no associations of rs41279104 were found either with the occurrence or with the degree of ED.

Although we did not find any association in the haplotype analysis of *NOS1* polymorphisms related to the degree of ED or to nitrite levels, curiously we found there were carriers of the TT haplotype (rs41279104 and rs2682826) only in the group with clinical ED (Supplementary Table S4). We know that the rs41279104 variant can directly imply the reduction of *NOS1* mRNA transcriptional activity by up to 30–50% in the prefrontal cortex [48,55] and negatively implicate functioning in the same brain region in schizophrenic patients [53]. In relation to rs2682826 carriers, an association of this variant with environmental factors in Parkinson’s disease can be seen [56], however another study shows that there is no association of this variant with the adverse effect of pharmacological treatment in Parkinson’s disease [57]. A better investigation in larger populations, both on cardiovascular conditions and other diseases, is necessary to show whether carriers of this *NOS1* TT haplotype may be more vulnerable to certain types of diseases or not.

Epidemiological studies have investigated the association of NOS gene variants with cardiovascular system phenotypes. Associations were found between *NOS1* variations with hypertension [49] and changes in blood pressure [50]. Moreover, a genome-wide association study (GWAS) for ischemic stroke identified *NOS1* as a strong candidate gene [58]. Ultimately, it is worth highlighting the importance of our results in the context of cardiovascular diseases, as the relationship between ED and CVDs is clear, especially regarding the risk of developing both.

The gene *PDE5A*, which encodes PDE5A, is located on chromosome 4, specifically at region 4q26, and alternative splicing of it results in three transcript variants encoding distinct isoforms [59]. The individuals carrying a variant of *PDE5A* polymorphisms (rs12646525 and rs3806808) were associated with a poorer response to Sildenafil treatment in patients with erectile dysfunction [60]. In addition, dogs carrying the *PDE5A* variant in the region of the exon that switches glutamic acid to lysine showed that when this variant is present, the animals tend to have lower levels of circulating cGMP [61]. The increase in circulating cGMP directly impacts erectile function, increasing the duration of penile erection, which is important for prolonged maintenance of sexual activity for people with ED [20,62]. Unfortunately, these data do not corroborate the present study where *PDE5A* polymorphisms were not associated with nitrite levels. As nitrite is a metabolite of NO in the organism, high levels of nitrite indicate greater bioavailability of NO. One study showed that diabetic patients with ED have a lower level of nitrite in the corpus cavernosum penis when compared only to ED [63]. In humans, it was also seen that an endogenous inhibitor of nitric oxide synthase (called ADMA) has a negative association with blood nitrite levels, indicating that in disease conditions there is an increase in the level of the endogenous NOS inhibitor (ADMA), contributing to a decrease in the formation of nitrite in the organism [64]. In this way, we can see the increase in nitrite as an indicator of NO production in the organism that can be beneficial for ED. Decreased PDE5A activity in vivo and in vitro located in the caveolae of the vascular endothelium can improve eNOS function by maintaining local homeostasis [65]. We know that the integrity of the vascular endothelium is also crucial for maintaining erectile function in men, as it synthesizes NO through eNOS [66].

Sildenafil, a PDE5 inhibitor, which is used as a first-choice drug for the treatment of ED, shows good efficacy for patients with ED [11]. Under physiological conditions, penile erection begins with the stimulation of non-adrenergic and non-cholinergic fibers (NANC), which leads to the production of NO [7]. Under these conditions, NO is synthesized through nNOS and L-arginine [15]. NO rapidly diffuses into the smooth muscle of the corpus cavernosum, which results in a decrease in intracellular Ca+ through GCs/cGMP pathways and protein kinase activation [7]. This process increases the local blood flow, consequently increasing the shear stress, which leads to the maintenance of the penile erection through the synthesis of NO by the enzyme eNOS derived from the vascular endothelium [19]. The end of the smooth muscle relaxation process of the corpus cavernosum occurs through the degradation of cGMP through the action of PDE5 [15]. Thus, when we look at all the mechanisms of penile erection, we can see that the polymorphisms studied here (*NOS1* and *PDE5A*) are part of this mechanism and require greater attention to better understand the vulnerability and risk that individuals may face in the future. 

As a strength of this study, we cite the careful phenotyping of clinical ED patients. Our exclusion criteria enriched the sample with vasculogenic ED and diabetes (with both vascular and neuronal damage). Thus, other possible confounding sources for ED were excluded, refining the evaluated phenotype. As limitations, we cite the low number of included participants, however, it is enough to provide clinically significant evidence as demonstrated in the power calculations, and the blood collection was performed in an anatomical location that may not reflect the levels of nitrite in the cavernous tissue, therefore it is possible that small effects after acute stimuli associated with penile erection may not have been detected when considering systemic evaluations.

Mechanistic investigation in specific tissues, such as the corpus cavernosum of the penile variation of the *NOS1*, and *PDE5A* polymorphisms studied here may bring new insights to confirm, for example, whether the association of the *NOS1* variant (rs2682826) related to the degree of ED found in this study, could be transferred to clinical practice to predict the severity of ED. This data could contribute with preventive treatments, as well as a personalized treatment of patients with ED.

## 5. Conclusions

The variant Ct and TT genotypes of the rs2682826 polymorphism are associated with more intense symptoms of erectile dysfunction, in other words, men with these genotypes have worse erectile function when compared to the ancestral CC genotype of this same polymorphism. This result may be useful in establishing a biomarker panel for ED risk and disability prediction, and for personalized therapy for ED.

## Figures and Tables

**Table 1:** Clinical features of the Control and Clinical ED groups.

	Control	Clinical ED	*p*
n	114	119	-
Age (years)	47 ± 9	56 ± 11	<0.001 *
Ethnicity (White/non-White)	61/53	56/63	0.325
Waist circumference (cm)	97 ± 11	100 ± 12	0.062
BMI (kg m^−2^)	28 ± 4	28 ± 5	0.643
*Smoking*			
Never smoker (n)	66	43	<0.001 *
Ex-smoker (n)	28	61	
Current smoker (n)	20	15	
Ethanol consumption			
Current HD (> 30 g/day)	11	5	0.009 *
Former HD (> 30 g/day)	4	16	
Low dose (<30 g/day)	99	98	
SBP (mm Hg)	131 ± 19	139 ± 19	0.001 *
DBP (mm Hg)	88 ± 13	89 ± 13	0.618
HDL (mg dL^−1^)	36 ± 11	41 ± 9	<0.001 *
LDL (mg dL^−1^)	127 ± 38	112 ± 35	0.002 *
Total cholesterol (mg dL^−1^)	206 ± 46	181 ± 38	<0.001 *
Triglycerides (mg dL^−1^)	175 ± 106	161 ± 111	0.323
Fasting glucose (mg dL^−1^)	100 ± 40	127 ± 55	<0.001 *
IIEF-5 score	28 ± 2	10 ± 7	<0.001 *

ED: erectile dysfunction; SBP: systolic blood pressure; DBP: diastolic blood pressure; HDL: high density lipoprotein cholesterol; LDL: low density lipoprotein cholesterol; IIEF-5: 5-item version of the international index of erectile function score. HD: high dose. Results are expressed as means ± SD or as absolute numbers. Groups were compared using chi-squared tests, Student’s t test or Mann Whitney tests, as appropriate. * Statistically significant.

**Table 2:** Multivariate linear regression analysis showing the association of genotypes of *NOS1* with IIEF scores on Control and Clinical ED groups.

Source	Control		Clinical ED	
	R² = 0.11	RMSE = 0.03	R² = 0.14	RMSE = 0.36
	B	*p*	B	*p*
Age (years)	−0.10	0.008 *	−1.11	0.006 *
Diabetes (yes)	+0.00	0.563	−0.00	0.918
*Smoking Status*				
Never Smoker	+0.00	0.837	−0.01	0.878
Ex Smoker	+0.00	0.379	+0.10	0.047 *
Current Smoker	−0.01	0.336	−0.09	0.173
Ethanol Consumption (>30 g/day)	−0.00	0.634	+0.04	0.664
rs41279104				
	B	*p*	B	*p*
CC	+0.00	0.662	−0.01	0.728
CT + TT	−0.00	0.662	+0.01	0.728
rs2682826				
	B	*p*	B	*p*
CC	−0.01	0.122	+0.07	0.033 *
CT + TT	+0.01	0.122	−0.07	0.033 *

ED: erectile dysfunction. R^2^: portion of variability explained by the model. RMSE: root mean square error. All continuous variables were log-normalized. * Statistically significant.

**Table 3:** Multivariate linear regression analysis showing the association of genotypes of *PDE5A* with IIEF scores on Control and Clinical ED groups.

Source	Control		Clinical ED	
	R² = 0.09	RMSE = 1.11	R² = 0.14	RMSE = 6.74
	B	*p*	B	*p*
Age (years)	−0.03	0.008	−0.21	<0.001
Diabetes (yes)	−0.05	0.836	−0.77	0.209
*Smoking Status*				
Never Smoker	−0.17	0.277	+0.97	0.281
Ex Smoker	−0.09	0.643	+0.94	0.292
Current Smoker	+0.27	0.231	−1.91	0.097
Ethanol Consumption (>30 g/day)	−0.15	0.418	−0.46	0.660
Genotypes				
rs2389866	B	*p*	B	*p*
TT	+0.06	0.706	+0.48	0.542
CT + CC	−0.06	0.706	−0.48	0.542
rs3733526	B	*p*	B	*p*
GG	+0.13	0.621	+0.01	0.997
AG + AA	−0.13	0.621	−0.01	0.997
rs13124532				
CC	+0.08	0.678	−0.83	0.402
CT + TT	−0.08	0.678	+0.83	0.402

ED: erectile dysfunction. R^2^: portion of variability explained by the model. RMSE: root mean square error. All continuous variables were log-normalized.

## Data Availability

Data is available upon request to the corresponding author.

## Supplementary Materials

The following supporting information can be downloaded at: https://www.mdpi.com/article/10.3390/life13051082/s1, Table S1: Genotypes distribution of rs41279104 and rs2682826 polymorphisms of *NOS1* and association with Erectile Dysfunction; Figure S1: IIEF versus genotypes of *NOS1* polymorphisms—Clinical ED group; Table S2: Multivariate linear regression analysis showing the association of genotypes of *NOS1* with nitrite levels on Control and Clinical ED groups; Table S3: Haplotypes distribution of *NOS1* polymorphisms and association with Erectile Dysfunction; Figure S2: IIEF versus haplotypes of *NOS1* polymorphisms—Clinical ED group; Table S4: Multivariate linear regression analysis showing the association of haplotypes of *NOS1* with IIEF scores on Control and Clinical ED groups; Figure S3: Nitrite plasma concentration versus haplotype of *NOS1* polymorphisms—Clinical ED group; Table S5: Multivariate linear regression analysis showing the association of haplotypes of *NOS1* with nitrite levels on Control and Clinical ED groups; Table S6: Genotypes distribution of polymorphisms rs2389866, rs3733526 and rs13124532 of *PDE5A* and association with Erectile Dysfunction; Figure S4: IIEF score versus genotypes of *PDE5A* polymorphisms—Clinical ED group; Table S7: Multivariate linear regression analysis showing the association of genotypes of *PDE5A* with nitrite levels on Control and Clinical ED groups; Table S8: Haplotypes distribution of polymorphisms rs2389866, rs3733526 and rs13124532 of *PDE5A* and association with Erectile Dysfunction; Figure S5: IIEF versus haplotypes *PDE5A* polymorphisms—Clinical ED group; Table S9: Multivariate linear regression analysis showing the association of haplotypes of *PDE5A* with IIEF scores on Control and Clinical ED groups; Figure S6: Nitrite plasma concentration versus haplotype of *PDE5A*—Clinical ED group; Table S10: Multivariate linear regression analysis showing the association of haplotypes of *PDE5A* with nitrite levels on Control and Clinical ED groups.
