# Supplementary material for: The rs2682826 Polymorphism of the NOS1 Gene Is Associated with the Degree of Disability of Erectile Dysfunction
Source: Life (Basel). 2023 Apr 25;13(5):1082. doi: 10.3390/life13051082 (PMC10221433; doi:10.3390/life13051082)
Supplement: Supplementary file 1 [file life-13-01082-s001.zip › life-2291661-supplementary.pdf]

Supplementary Table S1: Genotypes distribution of rs41279104 and rs2682826 polymorphisms of *NOS1* and association with Erectile Dysfunction.

**Polymorphism**	**Control (n=114)**	**Erectile Dysfunction (n=119)**	**P**	**OR (95% CI)**
rs41279104				
CC	91 (0.80)	91 (0.77)	–	1.00 (Reference)
CT + TT	23 (0.20)	28 (0.23)	0.536	1.22 (0.65 to 2.27)
Alleles				
C	204 (0.89)	206 (0.87)	–	1.00 (Reference)
T	24 (0.11)	32 (0.13)	0.333	1.32 (0.75 to 2.32)
rs2682826				
CC	59 (0.52)	61 (0.51)	–	1.00 (Reference)
CT + TT	55 (0.48)	58 (0.49)	0.940	1.02 (0.61 to 1.71)
Alleles				
C	165 (0.72)	173 (0.73)	-	1.00 (Reference)
T	63 (0.28)	65 (0.27)	0.938	0.98 (0.66 to 1.48)

OR, odds ratio; **95% CI**, 95% confidence interval. Data are expressed as n (frequency).

*: statistically significant

Supplementary Figure S1: IIEF versus genotypes of *NOS1* polymorphisms - Clinical ED group.

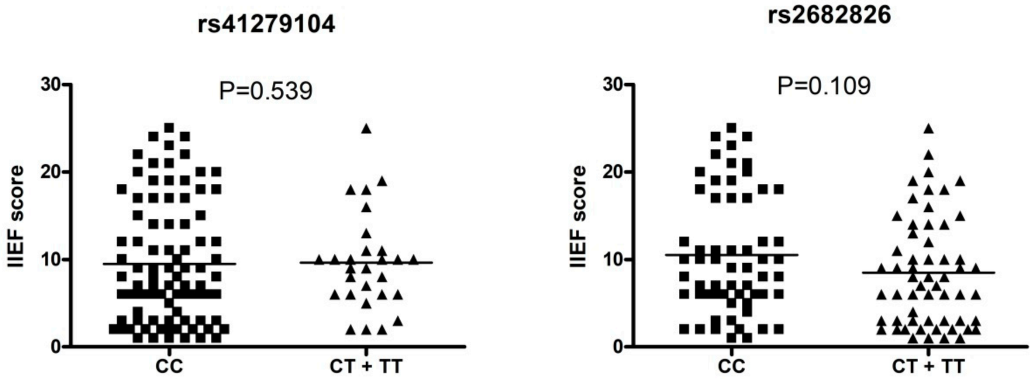

Values of the erectile function score expressed as mean ± standard deviation. Student’s t test.

Supplementary Table S2: Multivariate linear regression analysis showing the association of genotypes of *NOS1* with nitrite levels on Control and Clinical ED groups.

	Control		Clinical ED	
	R^2=0.05	RMSE=127	R^2=0.06	RMSE=183
Source	B	P	B	P
Age (years)	+0.01	0.988	+1.27	0.516
Diabetes (yes)	-21.3	0.209	-20.5	0.302
Smoking Status				
Never Smoker	+9.54	0.298	-41.2	0.167
Ex Smoker	-5.39	0.623	+27.1	0.326
Current Smoker	-4.15	0.728	+14.3	0.696
Ethanol Consumption (>30g/day)	+6.42	0.568	-16.6	0.711
Genotypes				
rs41279104	B	P	B	P
CC	+8.57	0.307	+11.1	0.631
CT + TT	-8.57	0.307	-11.1	0.631
rs2682826	B	P	B	P
CC	-7.53	0.253	+4.83	0.805
CT + TT	+7.53	0.253	-4.83	0.805

ED: erectile dysfunction. **R^2**: portion of variability explained by the model. **RMSE**: root mean square error. All continuous variables were log-normalized. TT haplotype was not observed in Control Group.

* Statistically significant.

Supplementary Table S3: Haplotypes distribution of *NOS1* polymorphisms and association with Erectile Dysfunction.

**Haplotype**	**Control (n=228)**	**Erectile Dysfunction (n=238)**	**P**	**OR (95% CI)**
CC	141	145	–	1.00 (Reference)
CT	63	61	0.780	0.94 (0.62 to 1.44)
TC	24	28	0.676	1.13 (0.63 to 2.05)
TT	0	4	0.050	8.75 (0.47 to 164)
			Global P = 0.242	

OR, odds ratio; **95% CI**, 95% confidence interval. Data are expressed as n (frequency).

*: statistically significant

Supplementary Figure S2: IIEF versus haplotypes of *NOS1* polymorphisms - Clinical ED group.

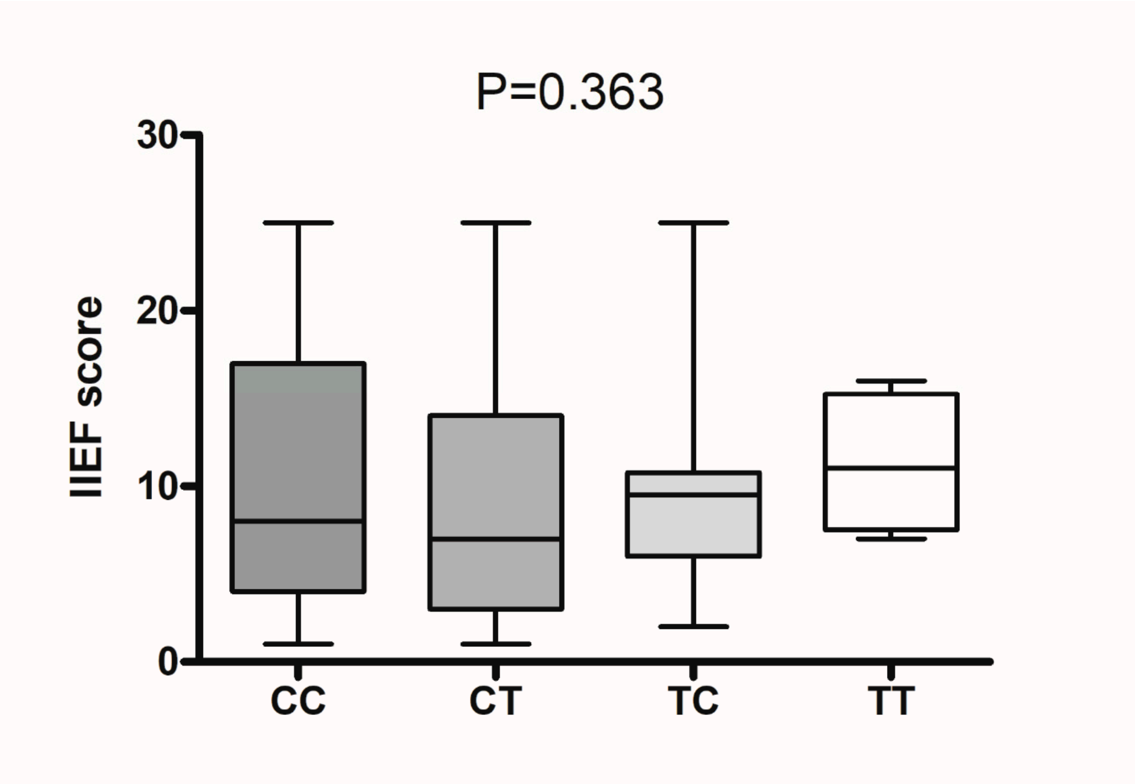

Values of the erectile function score expressed as median ± interquartile ranges. Kruskal-Wallis test with Dunn's post-test.

Supplementary Table S4: Multivariate linear regression analysis showing the association of haplotypes of *NOS1* with IIEF scores on Control and Clinical ED groups.

	Control		Clinical ED	
	R^2=0.09	RMSE=0.03	R^2=0.12	RMSE=0.36
Source	B	P	B	P
Age (years)	-0.10	<0.001*	-1.21	<0.001*
Diabetes (yes)	+0.00	0.385	-0.01	0.787
Smoking Status				
Never Smoker	+0.00	0.805	+0.00	0.942
Ex Smoker	+0.00	0.264	+0.09	0.010*
Current Smoker	-0.01	0.223	-0.09	0.050
Ethanol Consumption (>30g/day)	-0.00	0.413	+0.04	0.545
Haplotypes	P=0.212		P=0.351	
	B	P	B	P
CC	-0.00	0.446	+0.01	0.801
CT	+0.01	0.110	-0.07	0.188
TC	-0.00	0.483	+0.10	0.173
TT	-	-	-0.04	0.598

ED: erectile dysfunction. **R^2**: portion of variability explained by the model. **RMSE**: root mean square error. All continuous variables were log-normalized. TT haplotype was not observed in Control Group.

* Statistically significant.

Supplementary Figure S3: Nitrite plasma concentration versus haplotype of NOS1 polymorphisms - Clinical ED group.

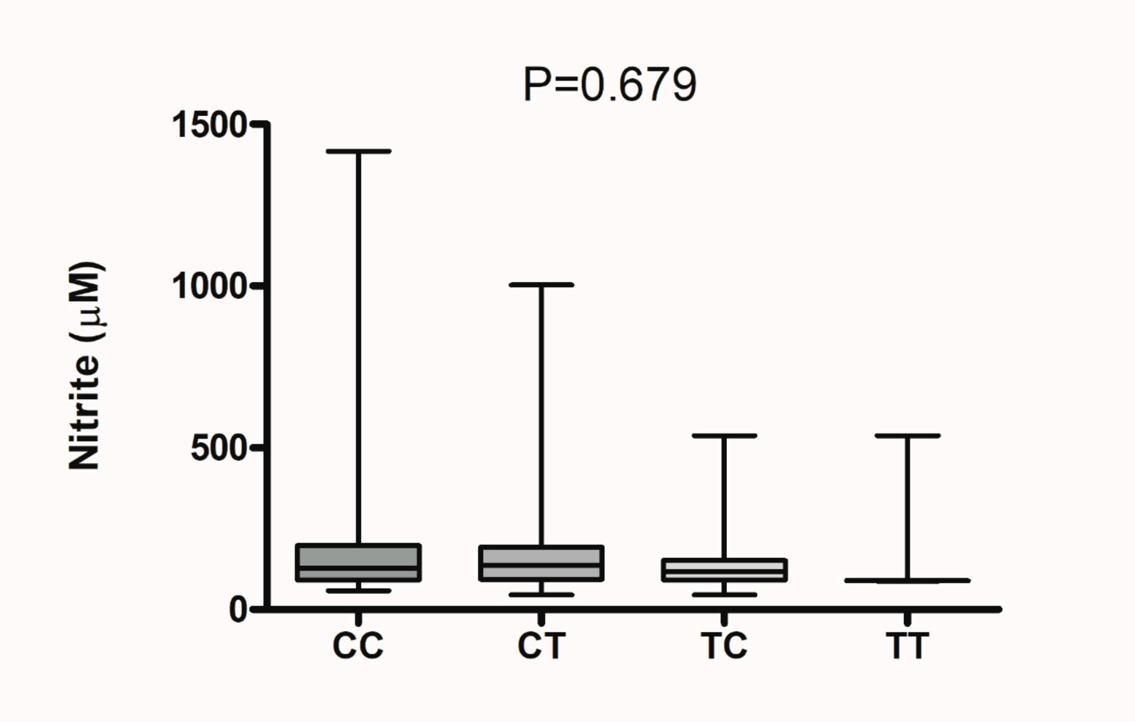

Values of the nitrite concentration expressed as median ± interquartile ranges. Kruskal-Wallis test with Dunn's post-test.

Supplementary Table S5: Multivariate linear regression analysis showing the association of haplotypes of *NOS1* with nitrite levels on Control and Clinical ED groups.

	Control		Clinical ED	
	R^2=0.04	RMSE=127	R^2=0.06	RMSE=177
Source	B	P	B	P
Age (years)	-0.02	0.966	+1.29	0.321
Diabetes (yes)	-20.0	0.088	-21.3	0.111
Smoking Status				
Never Smoker	+9.19	0.149	-42.4	0.035*
Ex Smoker	-6.60	0.384	+26.7	0.153
Current Smoker	-2.59	0.753	+15.6	0.531
Ethanol Consumption (>30g/day)	+5.76	0.462	-15.3	0.617
Haplotypes	P=0.176		P=0.823	
	B	P	B	P
CC	+0.27	0.967	+5.33	0.862
CT	+14.1	0.063	-19.4	0.574
TC	-14.4	0.149	-16.1	0.683
TT	-	-	+30.2	0.701

ED: erectile dysfunction. $\mathbf{R^2}$: portion of variability explained by the model. **RMSE**: root mean square error. All continuous variables were log-normalized. TT haplotype was not observed in Control Group.

* Statistically significant.

Supplementary Table S6: Genotypes distribution of polymorphisms rs2389866, rs3733526 and rs13124532 of *PDE5A* and association with Erectile Dysfunction.

**Polymorphism**	**Control (n=113†)**	**Erectile Dysfunction (n=115†)**	**P**	**OR (95% CI)**
rs2389866				
TT	73 (0.66)	71 (0.62)	–	1.00 (Reference)
TC + CC	38 (0.34)	44 (0.38)	0.529	1.19(0.69 to 2.05)
Alleles	222	230		
T	176 (0.79)	183 (0.80)	–	1.00 (Reference)
C	46 (0.21)	47 (0.20)	0.940	0.98(0.62 to 1.55)
rs3733526				
AA	79 (0.70)	78 (0.68)	–	1.00 (Reference)
AG + GG	34 (0.30)	36 (0.32)	0.808	1.07 (0.61 to 1.88)
Alleles	226	228		
A	185 (0.82)	188 (0.82)	-	1.00 (Reference)
G	41 (0.18)	40 (0.18)	0.868	0.96 (0.59 to 1.55)
rs13124532				
CC	91 (0.81)	96 (0.83)	–	1.00 (Reference)
CT + TT	22 (0.19)	19 (0.17)	0.562	0.82 (0.42 to 1.61)
Alleles				
C	201 (0.89)	209 (0.91)	–	1.00 (Reference)
T	25 (0.11)	21 (0.09)	0.494	0.81 (0.44 to 1.49)

OR, odds ratio; **95% CI**, 95% confidence interval. Data are expressed as n (frequency).

*: statistically significant; † Numbers may vary due to incomplete call rate in genotyping of *PDE5A*.

Supplementary Figure S4: IIEF score versus genotypes of *PDE5A* polymorphisms - Clinical ED group.

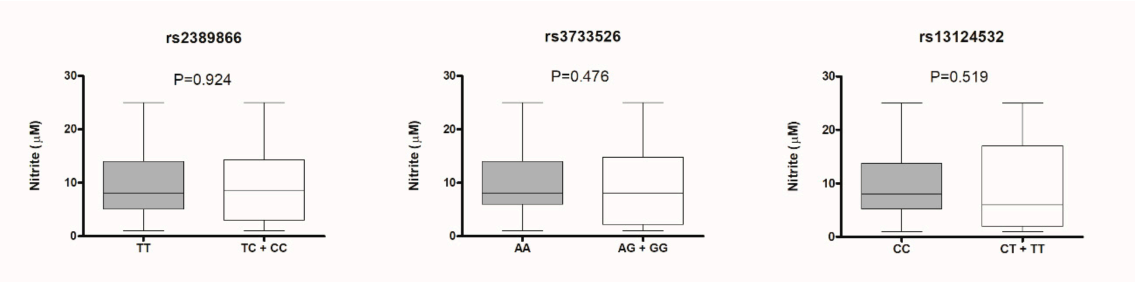

Values of the IIEF scores expressed as median ± interquartile ranges. Mann Whitney test.

Supplementary Table S7: Multivariate linear regression analysis showing the association of genotypes of *PDE5A* with nitrite levels on Control and Clinical ED groups.

	Control		Clinical ED	
	R^2=0.06	RMSE=70.5	R^2=0.11	RMSE=165.7
Source	B	P	B	P
Age (years)	-0.34	0.696	+1.58	0.288
Diabetes (yes)	-18.06	0.349	-14.74	0.361
Smoking Status				
Never Smoker	+10.29	0.338	-31.58	0.189
Ex Smoker	+1.49	0.910	+39.65	0.095
Current Smoker	-11.78	0.430	-8.07	0.791
Ethanol Consumption (>30g/day)	+7.65	0.559	+53.68	0.049
Genotypes				
rs2389866	B	P	B	P
TT	-14.71	0.185	+21.68	0.310
CT + CC	+14.71	0.185	-21.68	0.310
rs3733526	B	P	B	P
GG	-21.06	0.210	-11.51	0.776
AG + AA	+21.06	0.210	+11.51	0.776
rs13124532				
CC	+7.69	0.582	+14.80	0.571
CT + TT	-7.69	0.582	-14.80	0.571

ED: erectile dysfunction. **R^2**: portion of variability explained by the model. **RMSE**: root mean square error. All continuous variables were log-normalized.

* Statistically significant.

Supplementary Table S8: Haplotypes distribution of polymorphisms rs2389866, rs3733526 and rs13124532 of *PDE5A* and association with Erectile Dysfunction.

**Haplotype**	**Control (n=226)**	**Erectile Dysfunction (n=230)**	**P**	**OR (95% CI)**
TAC	175	179	–	1.00 (Reference)
TAT	1	0	0.313	0.33 (0.01 to 8.06)
TGC	4	4	0.978	0.98 (0.24 to 3.97)
CAC	9	11	0.670	1.20 (0.48 to 2.96)
CGC	13	15	0.759	1.13 (0.52 to 2.44)
CGT	24	21	0.622	0.86 (0.46 to 1.59)
			Global P = 0.907	

OR, odds ratio; **95% CI**, 95% confidence interval. Data are expressed as n (frequency).

*: statistically significant

Supplementary Figure S5: IIEF versus haplotypes *PDE5A* polymorphisms - Clinical ED group.

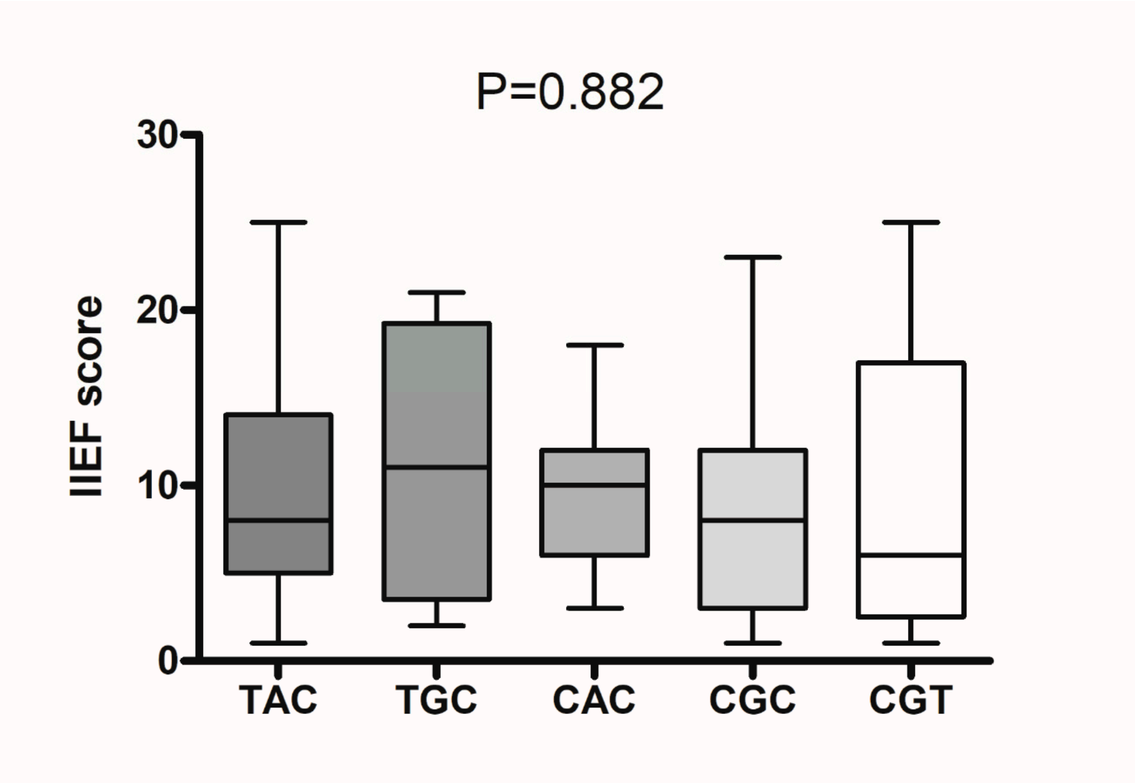

Values of the erectile function score expressed as median ± interquartile ranges. Kruskal-Wallis test with Dunn's post-test.

Supplementary Table S9: Multivariate linear regression analysis showing the association of haplotypes of *PDE5A* with IIEF scores on Control and Clinical ED groups.

	Control		Clinical ED	
	R^2=0.09	RMSE=1.81	R^2=0.20	RMSE=6.05
Source	B	P	B	P
Age (years)	-0.05	<0.001	-0.26	<0.001
Diabetes (yes)	+0.01	0.960	-0.32	0.494
Smoking Status				
Never Smoker	+0.24	0.200	-0.25	0.717
Ex Smoker	+0.02	0.920	+2.30	<0.001
Current Smoker	-0.27	0.278	-2.05	0.017
Ethanol Consumption (>30g/day)	-0.01	0.924	-0.79	0.194
Haplotypes	P=0.924		P=0.773	
	B	P	B	P
TAC	+0.08	0.791	-1.02	0.316
CGT	-0.10	0.811	-0.55	0.711
CGC	+0.34	0.502	+0.08	0.958
CAC	-0.49	0.477	-1.54	0.406
TGC	+0.17	0.818	+3.04	0.293

ED: erectile dysfunction. $\mathbf{R^2}$: portion of variability explained by the model. **RMSE**: root mean square error. All continuous variables were log-normalized.

* Statistically significant.

Supplementary Figure S6: Nitrite plasma concentration versus haplotype of *PDE5A* - Clinical ED group.

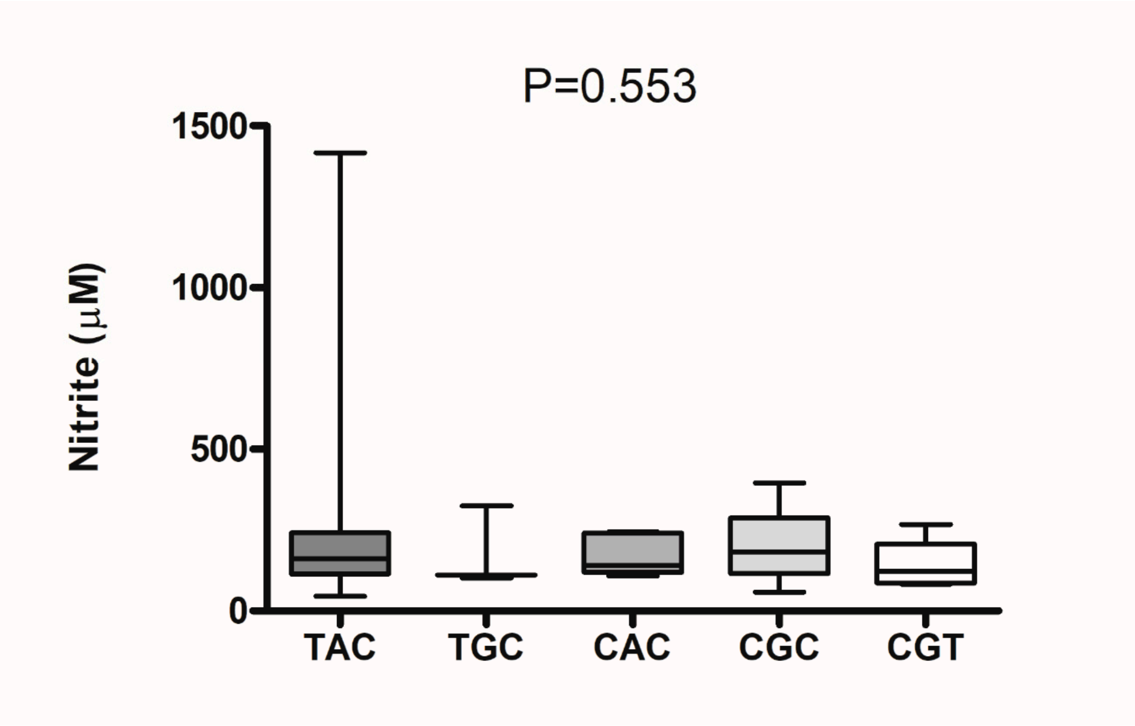

Values of the nitrite concentration expressed as median ± interquartile ranges.
Kruskal-Wallis test with Dunn's post-test.

Supplementary Table S10: Multivariate linear regression analysis showing the association of haplotypes of *PDE5A* with nitrite levels on Control and Clinical ED groups.

	Control		Clinical ED	
	R^2=0.03	RMSE=69.34	R^2=0.08	RMSE=178.86
Source	B	P	B	P
Age (years)	-0.29	0.624	+2.01	0.121
Diabetes (yes)	-24.19	0.086	-17.83	0.200
Smoking Status				
Never Smoker	+5.04	0.498	-37.90	0.067
Ex Smoker	-1.28	0.880	+29.23	0.128
Current Smoker	-3.75	0.695	+8.67	0.731
Ethanol Consumption (>30g/day)	+7.33	0.313	+30.50	0.092
Haplotypes	P=0.822		P=0.774	
	B	P	B	P
TAC	+4.66	0.694	+26.76	0.375
CGT	-6.75	0.688	-29.46	0.509
CGC	+15.76	0.422	-4.73	0.921
CAC	+11.50	0.666	-2.62	0.961
TGC	-25.18	0.388	+10.06	0.906

ED: erectile dysfunction. **R^2**: portion of variability explained by the model. **RMSE**: root mean square error. All continuous variables were log-normalized.

* Statistically significant.
